# Measuring COVID-19 vaccination coverage: an enhanced age-adjusted two-step floating catchment area model

**DOI:** 10.1186/s40249-021-00904-6

**Published:** 2021-09-16

**Authors:** Alireza Mohammadi, Abolfazl Mollalo, Robert Bergquist, Behzad Kiani

**Affiliations:** 1 Department of Geography and Urban Planning, Faculty of Social Sciences, University of Mohaghegh Ardabili, Ardabil, Iran; 2 Department of Public Health and Prevention Science, School of Health Sciences, Baldwin Wallace University, Berea, OH USA; 3 Ingerod, Brastad, Sweden (formerly with the UNICEF/UNDP/World Bank/WHO Special Programme for Research and Training in Tropical Diseases, World Health Organization), Geneva, Switzerland; 4 Department of Medical Informatics, School of Medicine, Mashhad University of Medical Sciences, Mashhad, Iran

**Keywords:** COVID-19, Spatial accessibility, Spatial inequality, Two-step floating catchment area, Vaccination coverage, Iran

## Abstract

**Background:**

There are only limited studies on access to COVID-19 vaccines and identifying the most appropriate health centres for performing vaccination in metropolitan areas. This study aimed to measure potential spatial access to COVID-19 vaccination centres in Mashhad, the second-most populous city in Iran.

**Methods:**

The 2021 age structure of the urban census tracts was integrated into the enhanced two-step floating catchment area model to improve accuracy. The model was developed based on three different access scenarios: only public hospitals, only public healthcare centres and both (either hospitals or healthcare centres) as potential vaccination facilities. The weighted decision-matrix and analytic hierarchy process, based on four criteria (i.e. service area, accessibility index, capacity of vaccination centres and distance to main roads), were used to choose potential vaccination centres looking for the highest suitability for residents. Global Moran’s index (GMI) was used to measure the spatial autocorrelation of the accessibility index in different scenarios and the proposed model.

**Results:**

There were 26 public hospitals and 271 public healthcare centres in the study area. Although the exclusive use of public healthcare centres for vaccination can provide the highest accessibility in the eastern and north-eastern parts of the study area, our findings indicate that including both public hospitals and public healthcare centres provide high accessibility to vaccination in central urban part. Therefore, a combination of public hospitals and public healthcare centres is recommended for efficient vaccination coverage. The value of GMI for the proposed model (accessibility to selected vaccination centres) was calculated as 0.53 (*Z* = 162.42, *P* < 0.01). Both GMI and *Z*-score values decreased in the proposed model, suggesting an enhancement in accessibility to COVID-19 vaccination services.

**Conclusions:**

The periphery and poor areas of the city had the least access to COVID-19 vaccination centres. Measuring spatial access to COVID-19 vaccination centres can provide valuable insights for urban public health decision-makers. Our model, coupled with geographical information systems, provides more efficient vaccination coverage by identifying the most suitable healthcare centres, which is of special importance when only few centres are available.

**Graphic abstract:**

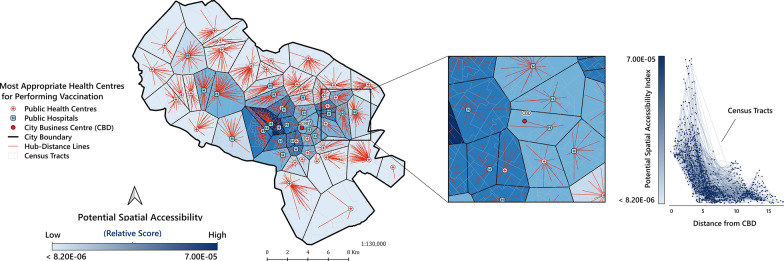

**Supplementary Information:**

The online version contains supplementary material available at 10.1186/s40249-021-00904-6.

## Background

The COVID-19 pandemic has imposed substantial costs on individuals and societies, both by direct impact on human physical and mental health, as well as indirectly through economic and social restrictions [1–3]. Non-pharmaceutical strategies such as social distancing, mask-wearing and economic lockdown are effective strategies halting transmission, but notoriously difficult to fully reinforce [4, 5, 6]. Several effective vaccines were developed, produced and passed regulatory offices in different countries a few months after the World Health Organization (WHO) declared the COVID-19 outbreak a global pandemic [7, 8]. Indeed, large-scale vaccination is considered the best strategy to address this crisis [9] and so far 12 different vaccines have been endorsed for full or restricted use by the WHO [10], and many countries are now striving to vaccinate their residents to reduce the risk. However, not only is vaccine production lagging demand [10], but access to vaccination centres is a hurdle making vaccine delivery a challenge; thus, careful spatial and logistic planning taking into account transport and storage (some vaccines require -80 °C), expiration dates, spacing between inoculations, inability to refreeze thawed vaccines, etc.) needed to ensure that everyone has appropriate access to vaccination against this new virus.

Access to healthcare is a question of the degree of effort needed to reach required medical services [11]. It has five primary dimensions: availability, accessibility, accommodation, affordability and acceptability [12, 13]. While availability refers to the number of available services in the healthcare centres [14], and it is evident that each healthcare facility cannot provide all different services that might be sought, we focussed on accessibility, i.e. the physical distance between healthcare centres and those who might need their services. This is ordinarily given by the length of, and how close to, the Euclidean distance (the straight line between source and destination). The real distance, i.e. the accessibility dimension, must be calculated considering all possible road connections available [15, 16], e.g., the drive time from an individual’s home to the healthcare centre [17–19]. As accessibility is related to geographical factors it is also labelled spatial access with affordability, accommodation and acceptability considered non-spatial access dimensions, while the availability dimension falls somewhere in-between [20, 21]. Another classification categorises access into revealed access and potential access, where the former refers to the actual use of services, while the latter is a proxy of the ability of individuals using these services [22]. In this study, access to COVID-19 vaccination considers the potential spatial access (PSA) to health centres (or hospitals) that includes the degree of geographical access considering both the geographical distance and the capacity to negotiate that distance.

As shown by our research group previously, the two-step floating catchment area (2SFCA) is a robust methodology to measure PSA to healthcare services. The method consists of two major steps. First, it calculates the capacity-to-population ratio for each healthcare location. Second, it sums the ratios for residential sites where healthcare locations overlap. However, the 2SFCA approach has drawn sharp criticism for disregarding the differences in accessibility within catchment areas assuming that all humans located within them have equal accessibility [23, 24]. To address this issue, the enhanced 2SFCA (E2SFCA) was developed by Lou and Qi [25] and has been further worked out by assigning geographical weights to both steps of the calculation process, which differentiates the travel-time zones through incorporating what is called distance-decay [26].

A limited number of studies have examined the spatial accessibility of people to COVID-19 vaccination facilities. However, an accessibility study of a centre proposed as a pilot COVID-19 vaccination programme in Hamilton, Ontario, Canada found that the selected sites did not serve the rural and urban residents appropriately; moreover, the associated cost of travel time was anticipated to be disproportionally borne by lower-income urban populations and rural residents [27]. Another study conducted in China compared four optimal vaccine distribution scenarios, including random access and strategies based on age, space as well as space and age strategy together and found that 30–40% vaccine coverage was needed to control the epidemic under the space and age strategy, while 60–70% vaccine coverage was required for the random access strategy [28]. Further, a Polish study conducted in the city of Warsaw measured spatial access to COVID-19 vaccination sites using Thiessen polygons (also known as Voronoi polygons) [29]. They identified spatial inequalities and areas with poor access to vaccination sites and proposed activating additional sites, either located ad hoc or using mobile vaccination sites to achieve uniform vaccination coverage. Importantly, the accessibility measurement model considered people aged 50–70, because they were either being vaccinated or would soon be [29]. A study in Florida, USA, evaluated the spatial accessibility to COVID-19 testing sites using the 2SFCA method by integrating both driving and walking modes. Their results suggest that increased efforts are needed to improve accessibility to testing sites among the elderly and those without private vehicles [30]. Another Florida study assessed the spatial accessibility of COVID-19 patients to intensive care unit (ICU) beds, using both the 2SFCA and the E2SFCA methods [31]. They developed an accessibility ratio difference index to evaluate the difference between the models based on spatial access and found that the 2SFCA method overestimated the accessibility in areas with a lower number of ICU beds due to the “equal access” assumption of the population within the catchment area (CA) [31].

A study in Brazil measured the geographic access to COVID-19 healthcare services using a balanced float CA approach and identified substantial social and spatial inequalities in access to health services during the pandemic [32]. Their findings indicated that ICU equipment availability varied considerably between cities and was substantially lower among Black communities and those of the poor [32]. Bauer et al. [33] measured access to ICU beds in 14 European countries using a regional ratio of ICU beds to 100 000 population as the accessibility index and the distance to the closest ICU and arrived at high indices in Germany, Estonia and Austria, with the lowest in Sweden and Denmark. Importantly, this study identified a negative correlation (*r* = -0.57*; P* value < 0.001) between ICU accessibility and the COVID-19 case fatality ratio [33]. Another study, conducted in Melbourne, Australia, incorporated the travel time from priority areas to palliative medicine and hospital services as estimates of accessibility thereby identifying the most vulnerable areas with respect to COVID-19 to be those where people were generally ≥ 65 years old and/or where people lived with disabilities [34]. While a study in Colombia found a high degree of spatial heterogeneity for ICU supplies in the study area by employing the E2SFCA to evaluate available ICU supplies for every thousand inhabitants in the Manizales-Villa María metropolitan area during the COVID-19 pandemic [35], results based on the same technique in Chicago, USA suggest that southern Chicago needs additional health care resources and that vulnerable populations often resided in areas with too low spatial accessibility [36].

With respect to COVID-19 vaccination, it is vital to prioritise the elderly population as it well known that higher age increases the risk of mortality [37, 38]. In this study, we measured the PSA to vaccination centres by developing a modified version of the E2SFCA model using a weighted population classified by age structure in each geographical area, as this enhancement should generate more realistic results for healthcare decision-making. A geographical information system (GIS)-based approach was used to choose the most appropriate potential healthcare centres for performing COVID-19 vaccination in an urban area.

## Methods

### Study area

**Fig. 1:**
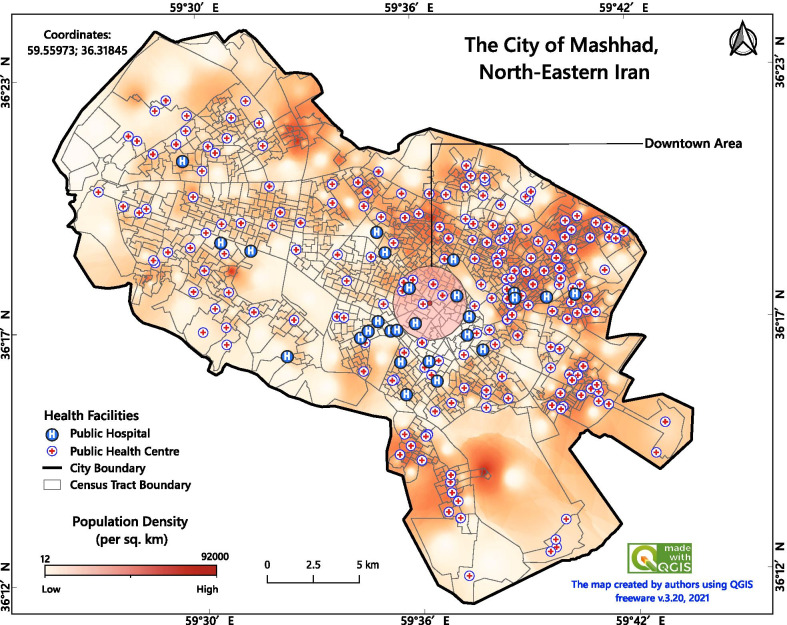
Geographic location of the study area, with distribution of hospitals, public healthcare centres and population density per km^2^

The methodology scheme is summarised in Additional file 1. The study location was the city of Mashhad, capital of Razavi-Khorasan Province in north-eastern Iran. Mashhad is located between latitudes 36°10’and 36°25’N, and longitudes 59°25’and 59°46’E, covering an area of 307 km^2^ (Fig. 1). According to the 2016 national census statistics, the city population was almost 3.3 million then and just slightly higher at the time of the study [39]. Mashhad consists of 17 municipality regions, 175 districts and 1301 census tracts (CTs). In this study, we considered the CTs as they provide the finest resolution for accessibility analysis. The average population density of CTs were 20 052 $\pm$ 10 983 individuals per km^2^. At the time of conducting this study, there were 26 active public hospitals and 271 public healthcare centres in Mashhad (Fig. 1).

### Data

Data on the 26 public hospitals and 271 public healthcare centres, including capacity that depends on available equipment and staff, were obtained from Mashhad University of Medical Sciences. Demographic characteristics of all CTs (including population size stratified by age and area) were obtained as a GIS shapefile from the Statistics Centre of Iran. The addresses of all healthcare centres and hospitals, city boundary and road network were retrieved from Mashhad Municipality and updated based on the Google Maps (https://www.google.com/maps) and OpenStreetMap (https://www.openstreetmap.org/) websites. All above data are freely available for public use via the link provided in the data availability section at the end of the article.

### Development of the age-integrated E2SFCA

#### Calculating the weighted population

To measure the PSA to vaccination centres, the age structure of each CT was integrated into the E2SFCA method as a local influential factor of COVID-19 mortality. However, we weighted each age group in the accessibility formula according to United States Centers for Disease Control and Prevention (CDC) in Atlanta, GA, USA [40]:

1 $$\begin{gathered} Weighted\,population = \hfill \\ Pop_{0 - 4} \times 1 + Pop_{5 - 17} \times 1 + Pop_{18 - 29} \times 10 + Pop_{30 - 39} \times 45 + Pop_{40 - 49} \times 130 + Pop_{50 - 64} \hfill \\ \times 440 + Pop_{65 - 74} \times 1300 + Pop_{75 - 84} \times 3200 + Pop_{ > = 85} \times 8700 \hfill \\ \end{gathered}$$

where *Pop*_*x*_ denotes the population for age group *x.* The age-related weights were applied, using ArcGIS v.10.8 software (ESRI, Redlands, CA, USA) to the CTs layer.

#### CA identification

The CA is the basis of the E2SFCA method [25]. According to previous studies [18, 24, 26, 41], we defined these areas by radius as 1 km (CA-1), 1.5 km (CA-2) and 2 km (CA-3) taking into account the average population density of the city (~ 20 000 people per km^2^). The distances chosen were the routine minimum and maximum values for defining service areas for health facilities in Iran’s major cities [42]. Moreover, the speed limit of 30 km/h (based on the average speed when driving a car in the city) was considered the basis of the travel-time analysis. The buffer analysis tool in ArcGIS software was used to calculate the CAs. The network analysis tools in QGIS v.3.20 open-access software v.3.20 (https://qgis.org/en/site/forusers/download.html) were used to identify the service areas.

#### Accessibility calculations

In step 1, the CAs were set at 1, 1.5, and 2-km distance to the *j*^th^ healthcare location. We searched all weighted population locations (*k*) within the threshold travel-time zone (*D*_*r*_) from healthcare centre *j *(CA _*j*_) using the travel-time zone (catchment) as the area formed based on the farthest accessibility boundaries (threshold) of each CT centroid to each potential vaccination centre and indicated by the distance (km) or time elapsed. This threshold was plotted based on the closest distance to vaccination centres in kilometres and we computed the vaccination capacity-to-weighted population ratio *R*_*j*_ within the CAs using Eq. 2 below following previous studies [24, 43, 44]:

2 $${R}_{j}=\frac{{S}_{j}}{{\sum }_{k\in \left\{{d}_{kj}\in {D}_{r}\right\}}{P}_{k}{W}_{r}}=\frac{{S}_{j}}{{\sum }_{k\in \left\{{d}_{kj}\in {D}_{1}\right\}}{P}_{k}{W}_{1}+{\sum }_{k\in \left\{{d}_{kj}\in {D}_{2}\right\}}{P}_{k}{W}_{2}+{\sum }_{k\in \left\{{d}_{kj}\in {D}_{3}\right\}}{P}_{k}{W}_{3}}$$

where *P*_*k*_ is the population of the *k*^*th*^ CT falling within the CA *j* (d_kj_ $\in$ D_r_); *S*_*j*_ the vaccination capacity at healthcare centre *j*; *d*_*kj*_ the travel time between *k* and *j*; and *D*_*r*_ the r^th^ travel time zone (r $\in \{\mathrm{1,2},3\}$) within the CA in question. *W*_*r*_ represents the distance weight for the *r*^*th*^ travel-time within the CA calculated by the Gaussian function. The weights set (1.00, 0.68, 0.22) were used to capture the distance decay of access to the *j*^*th*^ healthcare centre.

In step 2, we searched all locations *j* for people in *i*^*th*^ CT within the 1, 1.5, and 2-km distance radius. Then, using Eq. 3 below, we summed up the vaccination capacity-to weighted population ratios *R*_*j*_ (calculated in step 1) for all CTs. The same distance weights derived from the Gaussian function were applied in different travel-time zones to account for the distance decay.

3 $$A_{i}^{F} = \sum\nolimits_{{j \in \left\{ {d_{ij} \le D_{r} } \right\}}} {R_{j} } W_{r} = \sum\nolimits_{{j \in \left\{ {d_{ij} \le D_{1} } \right\}}} {R_{j} } W_{1} + \sum\nolimits_{{j \in \left\{ {d_{ij} \le D_{2} } \right\}}} {R_{j} } W_{2} + \sum\nolimits_{{j \in \left\{ {d_{ij} \le D_{3} } \right\}}} {R_{j} } W_{3}$$

where ${A}_{i}^{F}$ represents the accessibility vaccination centre for the population at location *i*; *R*_*j*_ the vaccination capacity-to-weighted population ratio at healthcare centre *j* that falls within the CA of population centre *i* (*d*_*kj*_ $\in$ D_r_); and *d*_*ij*_ the travel time between *i* and *j.* The E2SFCA calculations were performed under three different scenarios as follows:

##### Scenario 1: Accessibility to public hospitals (PHs)

In many developing countries, including Iran, PHs act as general and special care facilities and the first point of contact when the patient is referred to specialist care [26]. Hospitals are usually well-equipped and can thus be used as public vaccination sites when needed. In this scenario, the PSA to PHs as a potential vaccination centre (PVC) was calculated considering all 26 active PHs (Fig. 1). Since not all employed at the hospital were qualified and available to perform vaccinations, according to hospitals’ official, only 4% of each hospital’s staff capacity (using an average capacity of 24.85 people as vaccinators per hospital) was entered into the accessibility measurement.

##### Scenario 2: Accessibility to public healthcare centres (PHCs)

The PHCs, also known as primary care centres, are the second main health facilities that can be used for public vaccinations during pandemics. In this scenario, 271 PHCs with a capacity of 1 to 5 people (with an average capacity of 2.05 people as vaccinators), were included in the E2SFCA model. The number of PHCs is almost 10 times higher than that of hospitals and they are well-dispersed across the city. Therefore, the PHCs have a stronger potential to act as vaccination centres.

##### Scenario 3: Joint accessibility to PHs and PHCs

In this scenario, all 26 PHs and all 271 PHCs were entered into the E2SFCA model. All E2SFCA method calculations to measure the PSA index were performed using ArcGIS v.10.8.

### Choosing the most appropriate centres for vaccination (the proposed model)

Many countries have used available public space for vaccination, e.g., cinemas, shopping malls, even outdoor areas such as football arenas and the like. However, we did not take this possibility into account since our primary aim was to evaluate the capability of the modified version of the E2SFCA model to measure the PSA to available medical centres. Since it is not feasible to equip and prepare any resource for public vaccination, especially in developing countries, PHs and PHCs with high scores were selected for administering vaccines based on accessibility index, service capability, distance to main roads and capacity as vaccination centre as follows:

#### Accessibility index

**Fig. 2:**
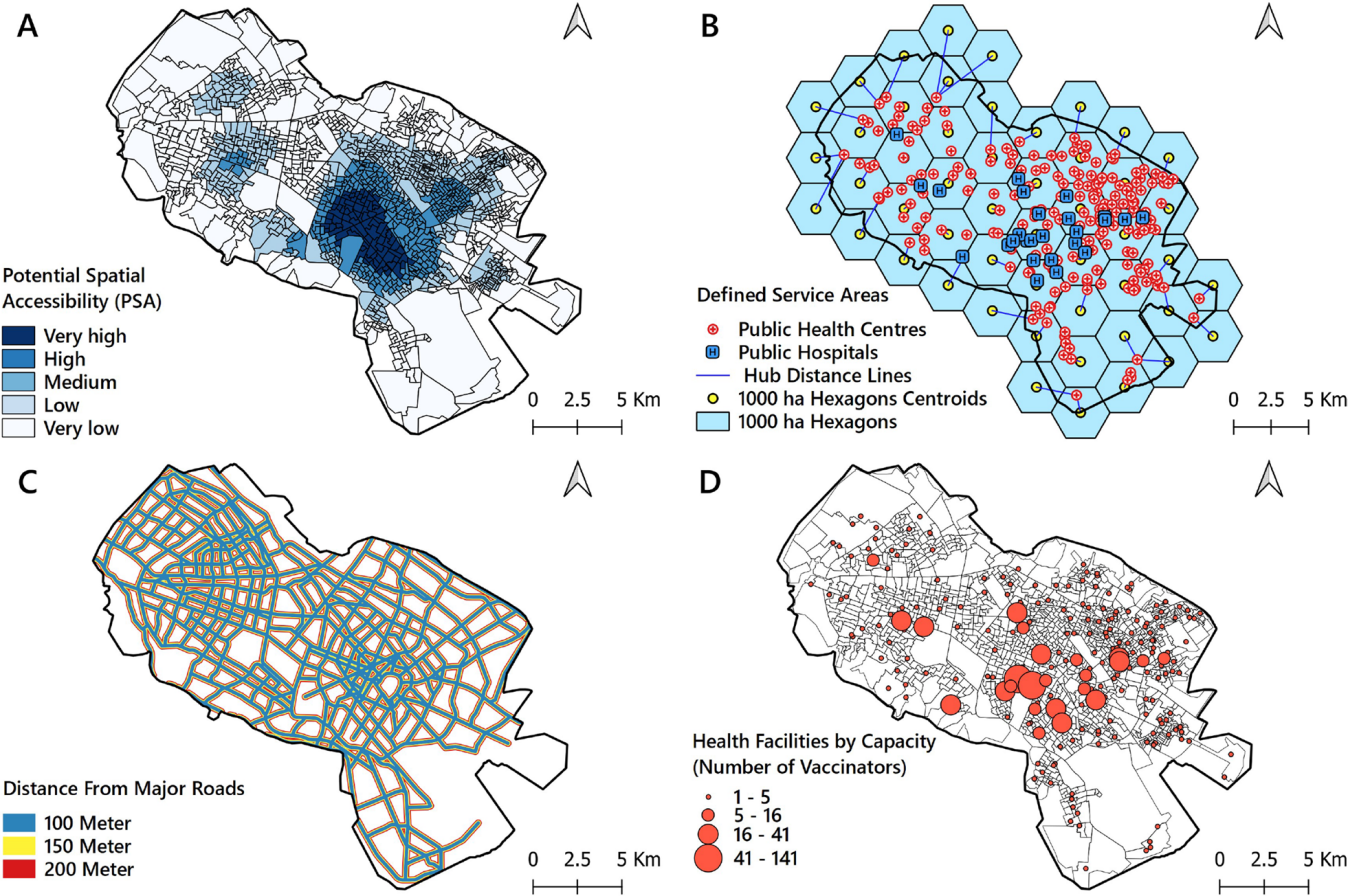
The criteria used to select potential COVID-19 vaccination centres. **A** PSA to PHs and PHCs. **B** user-defined equal-size hexagons as optimum service areas of potential vaccination centres; **C** distance from major roads; **D** capacity of potential vaccination centres

This index was derived from the calculation of PSA in scenario 3 (Fig. 2A) classified as five categories depending on the PSA for the following classes of CTs: very high (PSA = 2.50E−05 to 3.70E−05), high (PSA = 1.70E−05 to 2.50E−05), medium (PSA = 1.10E−05 to 1.70E−05), low (PSA = 6.00E−06 to 1.10E−05) and very low (PSA = 0 to 6.00E−06). The CTs falling into the very low category were considered as the highest priority for improved accessibility to vaccination. Therefore, higher weights were assigned to healthcare centres in CTs with low and very low PSAs. The weighting was employed to enhance the access to the nearest healthcare centre for people in deprived areas.

#### Service areas

Equitable accessibility requires optimum allocation of health facilities [45]. However, these areas do not have uniform geometric shapes, so the QGIS version of hexagonal tessellation was applied to achieve more homogeneous and geometrically defined service areas for the healthcare centres (depending on the area and population density of the city). The city was therefore divided into 50 equal 1000-ha hexagons (Fig. 2B). The radius of these theoretical service areas was set at 2 km, i.e. the maximum standard service area (coverage) for access to a health facility [46]. Then, the available PVCs closest to the geometric centres of these hexagons were given higher weights and consequently selected as centres with comparatively high suitability as vaccination sites.

#### Distance to main roads

Travel time and distance from the location of residents to health or medical facilities were the foundation when calculations were carried out to find locations with high accessibility [47]. In other words, medical facilities located at a convenient distance from main roads decrease travel time to the vaccination services, while larger distances to healthcare facilities (that translates into longer travel times) obstruct visits and revisits. There is no universally accepted superior relation between medical care facilities and the range of roads to reach it. According to Silalahi et al. [48], more attention should be paid to the transport network for easy access to PVCs. In this study, distances of 100 m (very desirable), 150 m (desirable) and 200 m (somewhat desirable) from the main roads were defined as one of the sets of criteria for selecting PVCs when applying buffer analysis in ArcGIS. The PVCs within the distance buffers were then assigned weights, and the PVCs outside these buffer zones with no access to main roads (especially for public transport users) were excluded from the analysis (Fig. 2C).

#### Vaccination centre capacity

In this study, PHs and then PHCs were ranked according to their capacity, so that centres with higher capacities were given a higher chance of selection. At the same time, the centres without proper facilities and equipment (e.g., centres with two vaccinators or less) were not given priority status (Fig. 2D).

The weighted decision-matrix method proposed by Pugh [49], practical both for simple and complex decisions [50], was used to select the most appropriate centres. First, a 297 × 4 matrix was formed. Then, each criterion was normalized through numbers between 0 and 1. According to the difference in importance of each main criterion, the weights were calculated using the analytical hierarchy process (AHP) [51] based on the opinions of 10 health professionals. Afterwards, the obtained weights from the main indices were multiplied by the corresponding numbers to arrive at the actual values of the selected criteria. For each potential vaccination centre, the obtained number from this calculation gave the final score that was finally used to rank the PVCs. The top 20% of the centres, i.e. those with the highest capability for vaccination according to the final scores, were selected. Business Performance Management, Singapore (BPMS) free web-based AHP online system [52] was used to calculate the weights of the various criteria.

### Accessibility to PVCs (reallocated centres)

After selecting the PVCs, the PSAs to these facilities were measured using the E2SFCA methodology.

### Evaluation of the model

Global Moran’s Index (GMI) was used to measure the spatial autocorrelation of the accessibility index in different scenarios and the proposed model. We assumed that a decrease in spatial autocorrelation of accessibility within CAs indicated improved accessibility. The GMI is defined by Eq. 4 [53]:

4 $$I = \frac{{n\left( {\mathop \sum \nolimits_{i = 1}^{n} \mathop \sum \nolimits_{j = 1}^{n} w_{ij} \left( {x_{i} - \overline{x}} \right)\left( {x_{j} - \overline{x}} \right)} \right)}}{{\left( {\mathop \sum \nolimits_{i = 1}^{n} \mathop \sum \nolimits_{j = 1}^{n} w_{ij} } \right)\left( {\mathop \sum \nolimits_{i = 1}^{n} \left( {x_{i} - \overline{x}} \right)^{2} } \right)}}$$

where *n* is the total number of spatial divisions (i.e. the CTs); x_i_ the value of the PSA in CT *i*; $\overline{x}$ the arithmetic mean for a given PSA; and *w*_*ij*_ the spatial weight matrix based on inverse distance and the Euclidean distance (distance band = 2983.7 m and the number of nearest neighbours = 4). The value of Moran’s *I* ranges from -1 to + 1. The further away it is from zero, the stronger (positive or negative) the autocorrelation [54]. ArcGIS v.10.8, software was used to compute the Moran’s index. A *P*-value < 0.01 was considered significant in the test using 99 permutations.

### Visualisation of the selected PVC areas of influence

This was done by Thiessen polygons, generated around a set of points in a given space by assigning all locations in that space to the closest member of the point set, a type of spatial tessellation called Voronoi diagrams [55]. Each polygon was created by QGIS and can be seen as an area of influence of a point in the given set [55]. The CTs were introduced as the origins and the PVCs as the destinations. In Fig. 4, the hub-distance (shown as red lines) indicates the distance (in km) from the centre of each CT (origin) to the nearest PVC (destination). The boundaries (in black) of the Thiessen polygons indicate the coverage of the service area of each PVC (Fig. 4A).

## Results

### Access to public hospitals as PVCs

**Fig. 3:**
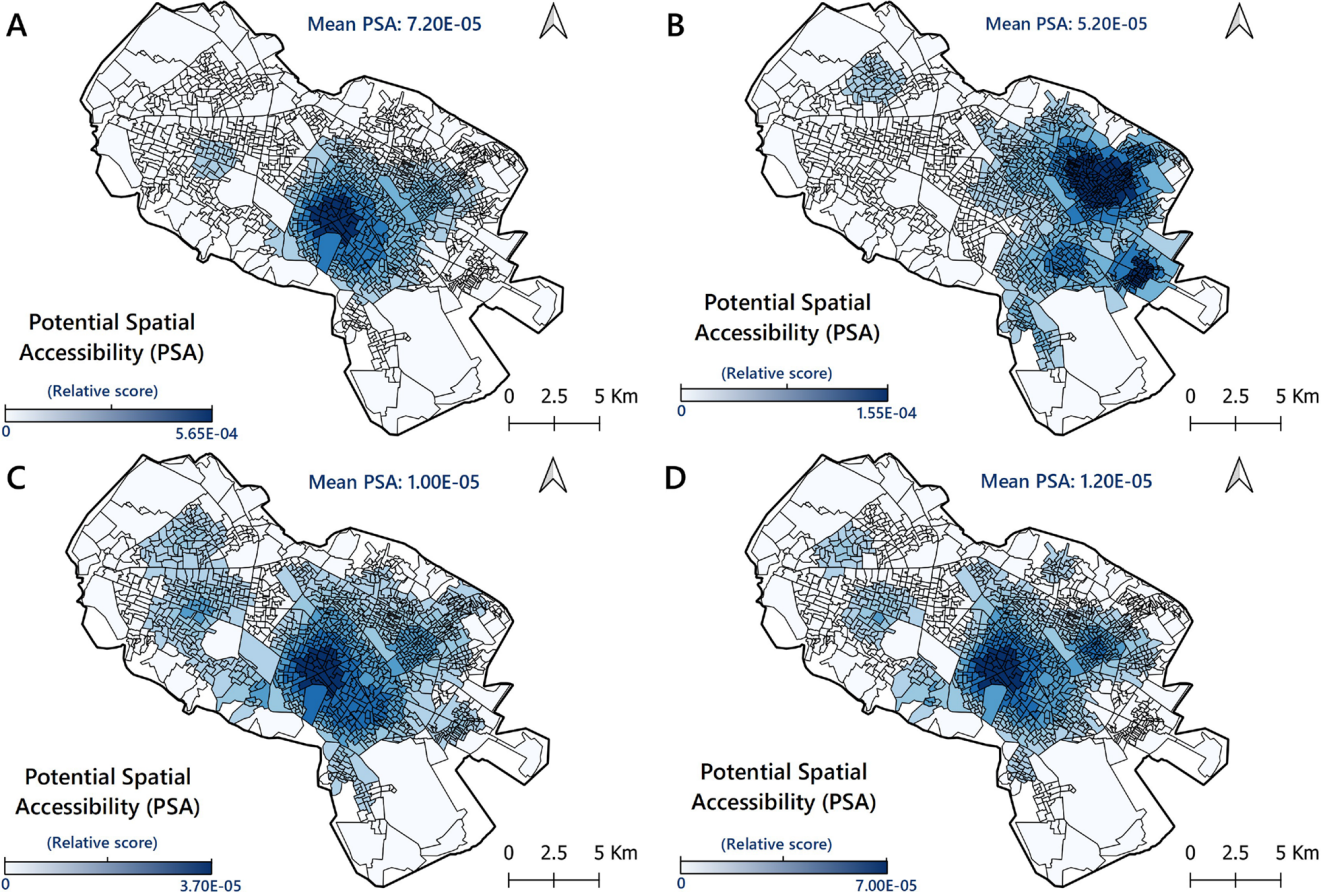
Spatial distribution of PSA to COVID-19 vaccination centre. **A** PSA to public hospitals; **B** PSA to public health centres; **C** PSA to public hospitals and public health centres; **D** PSA to the selected centres for performing vaccination

The results indicated that 864 CTs (66.4%) had low access to PHs (PSA < 7.20E−05) (Fig. 3A). Also, those CTs with above-average access to PVCs are often located in central parts of the city, where also most of the PHs (60%) are located. Moreover, the PSAs in 157 CTs (12.1%) were zero, mainly in the peripheral parts of the city and far from medical facilities and PHs.

### Access to public health centres

Out of the CTs, 768 (59.0%) had low access to the PHCs (PSA < 5.20E-05), which is illustrated by Fig. 3B. In only one CT, the PSA value was zero. This suggests that once the COVID-19 vaccine is available for all in all PHCs, most areas of the city would have access to vaccination services, but with low PSA values. In contrast, areas with higher-than-average PSA values were almost always found to be located in the eastern and north-eastern parts of the city. Hence, the spatial distribution of PSA values of the PHCs was not quite similar to the spatial distribution of PSA values of the PHs.

### Access to PHs and PHCs

The average value of PSA in the third scenario was 1.00E-05. According to Fig. 3C, 825 CTs (63.4%) had low access to these PVCs (PSA < 1.00E-05). Moreover, in 11 CTs (0.08%), the PSA value was zero. The CTs with higher PSA values were often seen to be located in central parts of the city.

### Access to selected PVCs (the proposed model)

Figure 3D depicts the results of the E2SFCA method for measuring access to the selected PVCs. Although only 60 centres out of 297 (20%) were included in the model, the average PSA was 1.20E-05, which indicates that 794 CTs (61.0%) have low access to PVCs (PSA < 1.20E−05). In 37 CTs (2.8%), the PSA value was zero. Moreover, the spatial distribution of PSA in this model was almost similar to the geographical pattern in the third scenario (i.e. the combination of PHS and PHCs). However, the PSA to PVCs was very low in the CTs located in all the suburban areas of the city. Table 1 shows the detailed results of PSA of three vaccination scenarios and the proposed model based on the E2SFCA method.

**Table 1:** Results of PSA index of three vaccination scenarios and the proposed model based on E2SFCA method

Scenario	PSA index	CTs (no.)	Share of CTs (%)
Scenario 1: Hospitals	Very low: 0 to 3.90E−05	719	55.27
	Low: 3.90E−05 to 1.09E−04	240	18.45
	Medium: 1.09E−04 to 2.04E−04	223	17.14
	High: 2.04E−04 to 3.90E−05	88	6.76
	Very High: 3.90E−05 to 5.65E−04	31	2.38
	Mean: 7.20E−05	-	-
Scenario 2: Health centres	Very low: 0 to 2.70E−05	440	33.82
	Low: 2.70E−05 to 4.80E−05	271	20.83
	Medium: 4.80E−05 to 7.40E−05	262	20.14
	High: 7.40E−05 to 1.08E−04	178	13.68
	Very High: 1.08E−04 to 1.55E−04	150	11.53
	Mean: 5.20E−05	-	-
Scenario 3: Hospitals & health centres	Very low: 0 to 6.10E−06	398	30.59
	Low: 6.10E−06 to 1.07E−05	471	36.20
	Medium: 1.07E−05 to 1.68E−05	284	21.83
	High: 1.68E−05 to 2.49E−05	117	8.99
	Very High: 2.49E−05 to 3.66E−05	31	2.38
	Mean: 1.00E−05	-	-
Proposed model: The top 20% of the PVCs	Very low: 0 to 7.50E−06	604	46.43
	Low: 7.50E−06 to 1.63E−05	315	24.21
	Medium: 1.63E−05 to 2.67E−05	233	17.91
	High: 2.67E−05 to 4.38E−05	120	9.22
	Very High: 4.38E−05 to 6.96E−05	29	2.23
	Mean: 1.20E−05	-	-

*PSA* Potential spatial accessibility, *CT* Census tracts, *PVC* Potential vaccination centre

### Model evaluation

Table 2 shows the summary statistics for spatial autocorrelation for the three scenarios and the proposed model. Moran’s *I* for the first scenario (PSA to PHs) was 0.6 (*Z* = 182.52, *P* < 0.01) indicating a clustered and unequal distribution of the PSA to hospitals in the city. The value of this index for the PSA for the second scenario (PSA to PHCs) was 0.75 (*Z* = 226.40, *P* < 0.01). For the third scenario (PSA to PHs and PHCs), this index was 0.57 (*Z* = 173.40, *P* < 0.01). Moran’s *I* and the *Z*-score values decreased in the third scenario, indicating a more uniform distribution of the PSA index compared to the first two scenarios. Finally, the value of GMI for the proposed model (PSA to selected PVCs) was calculated as 0.53 (*Z* = 162.42, *P* < 0.01). Both GMI and *Z*-score values decreased in the proposed model, suggesting an enhancement in PSA to COVID-19 vaccination services.

**Table 2:** Results of spatial autocorrelation (GMI) of three vaccination scenarios and the proposed model based on PSA

PSA	Global Moran Summary			
	Moran’s *I*	*Z*-score	*P*-value	Distribution pattern
Scenario 1: Hospitals	0.60	182.52	0.00	Clustered
Scenario 2: Health centres	0.75	226.40	0.00	Clustered
Scenario 3: Hospitals & Health centres	0.57	173.40	0.00	Clustered
Proposed model: Top 20% PVCs	0.53	162.42	0.00	Clustered

*PSA* Potential spatial access, *PVC* Potential vaccination centre

### Visualisation of the selected PVCs’ areas of influence

**Fig. 4:**
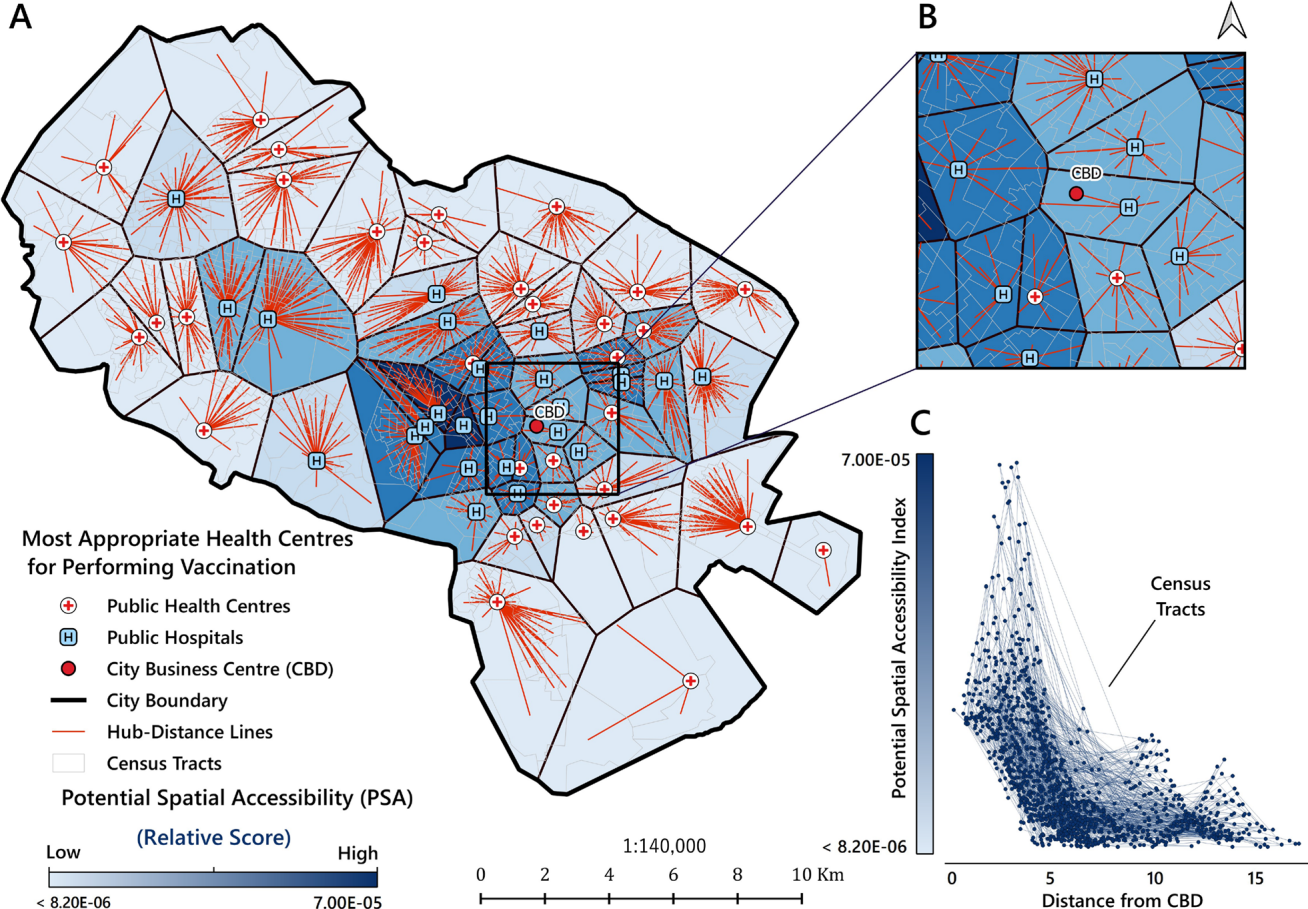
Proposed distribution for allocating potential COVID-19 vaccination centres. **A** areas of influence map of top 20% potential vaccination centres; **B** magnified window of map A focused on CBD area; **C** the relationship between PSA and distance from the CBD area. The strength of the blue colour Thiessen polygon ramp indicates the relative PSA score

Figure 4A shows the “areas of influence” (i.e. the service areas) of the selected PVCs based on Thiessen polygons. According to this map, each CT is connected to the nearest PVCs for vaccination services (average distance = 0.99 km). The analysis based on area of influence indicates that even though in the central parts of the city the PSA value is still high, all CTs across the city have access to at least one of the PVCs. Figure 4B depicts an overview (zoomed in) map of the Thiessen polygons in the Central Business District (CBD) of the city. According to Fig. 4C, the CTs located at a range distance of 0 to 5 km from the CBD, had the highest PSA values, while the PSA values decreased for the CTs far from the CBD.

## Discussion

We developed an application of the E2SFCA method for identifying the location of vaccinations centres by incorporating the CT population age structure to obtain a more realistic measure of PSA in the metropolitan area. Although the research methodology was developed for a specific place, Mashhad City in Iran, the methodology is replicable and can be applied in any urban area when vaccination supply, urban population, road network and the average driving speed is known. The PSA to PHs (first scenario) measurements showed low values of PSA for a large number of CTs. Additionally, the results of GMI showed a clustered distribution of PSA in this scenario because the hospitals are mainly concentrated in the city centre, with no homogeneous distribution across the study area. This should be considered when planning hospitals and health centres in rapidly growing developing countries, since an unequal distribution of hospitals with a concentration in the central metropolitan areas is currently common [26]. Consistent with the findings of Pereira et al. [32] in Brazil, our study found that there is an intense spatial inequality between downtown areas and marginal poor neighbourhoods with respect to access to PHs and PVCs. Our findings also suggest that a significant number of CTs around the city had virtually no access to COVID-19 vaccinations due to the dearth of hospitals there. However, although hospitals have a greater capacity for vaccination than any other health facility, this criterion alone is not sufficient to provide equitable access vaccination services in metropolitan areas. To reduce disparities and remedy this situation, public health policymakers should take into account all available vaccination places to defy uneven coverage.

Findings of PSA to PHCs (second scenario) showed that the average total accessibility to PHCs was lower than the average PSA to PHs due to a general lower PHC capacity. However, according to this scenario, it is possible to provide services for many CTs since the number of CTs accessing the PVCs was 7% higher than in the first scenario. Moreover, the results of GMI for this scenario showed that the PSA to PHCs had a highly clustered distribution, suggesting that these centres were not uniformly distributed in the city (they were in fact highly concentrated in the north-eastern areas). Therefore, this scenario may not be a realistic choice for providing spatial access to COVID-19 vaccination services. As stated by Jacobson et al. [56], many developing countries often encounter vaccine shortages, while they should be optimally distributed by the providers. Moreover, according to Shadmi et al. [57], despite the availability of vaccines in major cities of developing countries, many barriers can prevent spatial PVC access, such as uneven spatial distribution of health facilities and improper transportation systems.

PSA to both PHs and PHCs (third scenario) indicated that more CTs could receive appropriate vaccination services than in the first scenario. The GMI also showed that the spatial distribution of PSA tended towards a less clustered pattern compared to the first and second scenarios. This means that PVCs can be available to residents in a wider geographic area. The results support the notion that the number of CTs with PSA = 0 decreased to 0.84% compared to the first scenario in the third scenario, with the result that the third scenario stands out as providing more equitable spatial access. However, similar to the second scenario, it is impossible for government and local authorities to equip 297 centres. Therefore, this scenario cannot properly provide COVID-19 vaccination for a metropolitan area, especially not in developing countries with limited financial resources.

The PSA results of the proposed model indicates that, despite the selection of only 20% of high-capability centres as COVID-19 vaccination services, more CTs would have access to COVID-19 vaccination centres than in the first and third scenarios. Second, the GMI results show that the spatial distribution of PSA is less focused than the other scenarios, with a decreased strength of PSA clustering. This means a more equitable distribution of PVCs and effective criteria in selecting PVCs by improving the PSA. Despite selecting high-priority centres for vaccination, the PSA rate in the proposed model was still high in some areas, including the centre and the areas surrounding the CBD. In contrast, the rates were close to zero in other areas due to the high concentration of health facilities in metropolitan areas, as seen in too many developing countries [58]. Similar to what Zhao et al. [58] reports for Beijing, China, the public transportation system of our study area was found to be unevenly distributed, with most transportation facilities (e.g., buses and metro stations) concentrated in the city centre. According to Kenyon et al. [59], the obstacles associated with transportation can worsen disparities in access to health facilities such as vaccination centres. As expressed by Tseng et al. [60], supply capacity and the service area of a catchment in the E2SFCA method need to vary based on the type of supply. Therefore, we considered the number of vaccinators in this study as supply capacities to selected PVCs. Thus, to maximize service coverage, the defined service areas were considered for vaccine supply centres. These designated areas allowed us to choose the most accessible centres to provide vaccine services to all city areas. At the same time, the analysis results with reference to the areas of influence showed that despite restrictions to equitable access (< 1 km), all CTs had access to at least one available centre for performing COVID-19 vaccination. This 1-km accessibility radius can be suitable for metropolitan areas, especially in developing countries, during a short-time vaccination programme.

The limitations in this study are mainly associated with data quality. First, the road network dataset did not contain traffic information to apply multi-modal travel-time techniques. Second, the data for estimating the flow of residents during day and night were not available. Third, the mortality weights for each age group obtained via CDC were not sensitive to local parameters. In spite of the above-mentioned limitations, our results should contribute to pandemic‐related policymaking at the local level.

The findings of this study could assist policymakers’ long-term health planning as it would result in a more equitable distribution of primary healthcare facilities in large cities. As it is difficult to employ all health facilities for administrating COVID-19 vaccination in large cities, particularly in developing countries, quantifying the priority of the existing centres for performing vaccination against COVID-19 is inevitable. Further studies should consider dynamic and multi-modal travel-time methods to measure PSA, for example by the use of mixed indicators to select COVID-19 vaccination centres. In addition, as the COVID-19 vaccine is free of charge for all people, future research should focus on acceptability and accommodation components by addressing ways to improve vaccine availability for vulnerable populations. It is also suggested that future studies combine spatial and temporal components (i.e. working days and hours of health centres) for more realistic measure of accessibility to COVID-19 vaccine services.

### Research implications

- When choosing vaccination sites, it is necessary to use community health centres in addition to hospitals to decrease spatial inequality.
- To achieve more efficient COVID-19 vaccination, GIS can be used to quantify the suitability of existing healthcare centres in urban areas.
- Modelling of equitable COVID-19 vaccination services in metropolitan areas should not only include healthcare centre capacity, but also transportation networks and spatial access as they jointly influence the availability of vaccination.

## Conclusions

Our findings have important policy implications. The results show that the periphery and poor areas of the city had the least access to PVCs. Therefore, due to the large size of the study area and as it is common for people with lower socio-economic status to commute using public transportations, it is suggested to provide vaccination services in neighbourhoods with better access to public transportation.

The spatial accessibility models can measure the accessibility to potential vaccination services so that all individuals would have adequate and equitable access to COVID-19 vaccination services. We found that using urban indicators in selecting the most appropriate health facilities can help policymakers improve the accessibility to COVID-19 vaccination services in a cost-effective and timely fashion. In addition, the proposed approach in this study can easily be automated and broadly applied to various urban settings.

## Supplementary Information

**Additional file 1. The flowchart view of the methodology scheme in this study.**

## Data Availability

The datasets generated and/or analysed during the current study are available in the HARVARD Dataverse repository, [https://doi.org/10.7910/DVN/PNQUKX].

## Abbreviations

PSA: Potential spatial accessibility
2SFCA: Two-Step Floating Catchment Area
E2SFCA: Enhance Two-Step Floating Catchment Area
ICU: Intensive care unit
CA: Catchment area
CTs: Census tracts
GIS: Geographical information systems
PHs: Public hospitals
PVC: Potential vaccination centre
PHCs: Public health centres
AHP: Analytical hierarchy process

## References

[1] Azizi MR, Atlasi R, Ziapour A, Abbas J, Naemi R (2021). Innovative human resource management strategies during the COVID-19 pandemic: a systematic narrative review approach. Heliyon..

[2] Abbas J, Wang D, Su Z, Ziapour A (2021). The role of social media in the advent of covid-19 pandemic: Crisis management, mental health challenges and implications. Risk Manag Healthc Policy.

[3] Su Z, McDonnell D, Wen J, Kozak M, Abbas J, Šegalo S (2021). Mental health consequences of COVID-19 media coverage: the need for effective crisis communication practices. Glob Health.

[4] Askitas N, Tatsiramos K, Verheyden B (2021). Estimating worldwide effects of non-pharmaceutical interventions on COVID-19 incidence and population mobility patterns using a multiple-event study. Sci Rep.

[5] Liu Y, Morgenstern C, Kelly J, Lowe R, Jit M, Group CCW (2021). The impact of non-pharmaceutical interventions on SARS-CoV-2 transmission across 130 countries and territories. BMC Med.

[6] Wang Chunlei, Wang Dake, Abbas Jaffar, Duan Kaifeng, Mubeen Riaqa (2021). Global Financial Crisis, Smart Lockdown Strategies, and the COVID-19 Spillover Impacts: A Global Perspective Implications From Southeast Asia. Frontiers in Psychiatry.

[7] Bergquist R, Kiani B, Manda S (2020). First year with COVID-19: assessment and prospects. Geospat Health.

[8] Su Z, Wen J, Abbas J, McDonnell D, Cheshmehzangi A, Li X (2020). A race for a better understanding of COVID-19 vaccine non-adopters. Brain Behav Immun.

[9] Bloom DE, Cadarette D, Ferranna M, Hyer RN, Tortorice DL (2021). How new models of vaccine development for COVID-19 have helped address an epic public health crisis. Health Aff.

[10] Zimmer C CJ, Wee SL. Coronavirus vaccine tracker [Internet]: New York: The New York Times Company; 2020. https://www.nytimes.com/interactive/2020/science/coronavirus-vaccine-tracker.html. Accessed 25 Jun 2021.

[11] Hoseini B, Bagheri N, Kiani B, Azizi A, Tabesh H, Tara M (2018). Access to dialysis services: a systematic mapping review based on geographical information systems. Geospat Health.

[12] Saurman E (2016). Improving access: modifying Penchansky and Thomas's Theory of Access. J Health Serv Res Policy.

[13] Levesque JF, Harris MF, Russell G (2013). Patient-centred access to health care: conceptualising access at the interface of health systems and populations. Int J Equity Health.

[14] Gebremariam MK, Vaque-Crusellas C, Andersen LF, Stok FM, Stelmach-Mardas M, Brug J (2017). Measurement of availability and accessibility of food among youth: a systematic review of methodological studies. Int J Behav Nutr Phys Act.

[15] Tharumia Jagadeesan C, Wirtz VJ (2021). Geographical accessibility of medicines: a systematic literature review of pharmacy mapping. J Pharm Policy Pract.

[16] Apparicio P, Abdelmajid M, Riva M, Shearmur R (2008). Comparing alternative approaches to measuring the geographical accessibility of urban health services: Distance types and aggregation-error issues. Int J Health Geogr.

[17] Kiani B, Bagheri N, Tara A, Hoseini B, Tara M (2017). Haemodialysis services in the northeastern region of Iran. Geospat Health.

[18] Kiani B, Bagheri N, Tara A, Hoseini B, Tabesh H, Tara M (2017). Revealed access to haemodialysis facilities in northeastern Iran: factors that matter in rural and urban areas. Geospat Health.

[19] Azimi A, Bagheri N, Mostafavi SM, Furst MA, Hashtarkhani S, Amin FH (2021). Spatial-time analysis of cardiovascular emergency medical requests: enlightening policy and practice. BMC Public Health.

[20] Penchansky R, Thomas JW (1981). The concept of access: definition and relationship to consumer satisfaction. Med Care.

[21] Levesque JF, Harris MF, Russell G (2013). Patient-centred access to health care: conceptualising access at the interface of health systems and populations. Int J Equity Health.

[22] Kiani B, Bagheri N, Tara A, Hoseini B, Hashtarkhani S, Tara M (2018). Comparing potential spatial access with self-reported travel times and cost analysis to haemodialysis facilities in North-eastern Iran. Geospat Health.

[23] McGrail MR, Humphreys JS (2014). Measuring spatial accessibility to primary health care services: Utilising dynamic catchment sizes. Appl Geogr.

[24] Hashtarkhani S, Kiani B, Bergquist R, Bagheri N, VafaeiNejad R, Tara M (2020). An age-integrated approach to improve measurement of potential spatial accessibility to emergency medical services for urban areas. Int J Health Plan Manag.

[25] Luo W, Qi Y (2009). An enhanced two-step floating catchment area (E2SFCA) method for measuring spatial accessibility to primary care physicians. Health Place.

[26] Kiani B, Mohammadi A, Bergquist R, Bagheri N (2021). Different configurations of the two-step floating catchment area method for measuring the spatial accessibility to hospitals for people living with disability: a cross-sectional study. Arch Public Health.

[27] Paez A, Higgins CD (2021). The Accessibility Implications of a Pilot COVID-19 Vaccination Program in Hamilton.

[28] Zhou S, Zhou S, Zheng Z, Lu J (2021). Optimizing spatial allocation of COVID-19 vaccine by agent-based spatiotemporal simulations. GeoHealth.

[29] Krzysztofowicz S, Osińska-Skotak K (2021). The use of GIS technology to optimize COVID-19 vaccine distribution: a case study of the city of Warsaw, Poland. Int J Environ Res Public Health.

[30] Tao R, Downs J, Beckie TM, Chen Y, McNelley W (2020). Examining spatial accessibility to COVID-19 testing sites in Florida. Ann GIS.

[31] Ghorbanzadeh M, Kim K, Erman Ozguven E, Horner MW (2021). Spatial accessibility assessment of COVID-19 patients to healthcare facilities: a case study of Florida. Travel Behav Soc.

[32] Pereira RHM, Braga CKV, Servo LM, Serra B, Amaral P, Gouveia N (2021). Geographic access to COVID-19 healthcare in Brazil using a balanced float catchment area approach. Soc Sci Med.

[33] Bauer J, Bruggmann D, Klingelhofer D, Maier W, Schwettmann L, Weiss DJ (2020). Access to intensive care in 14 European countries: a spatial analysis of intensive care need and capacity in the light of COVID-19. Intensive Care Med.

[34] Lakhani A (2020). Which melbourne metropolitan areas are vulnerable to COVID-19 based on age, disability, and access to health services? Using spatial analysis to identify service gaps and inform delivery. J Pain Symptom Manag.

[35] Escobar DA, Cardona S, Ruiz S (2020). Planning of expansion of ICU hospital care in times of Covid-19 using the E2SFCA model. Rev Espacios..

[36] Kang JY, Michels A, Lyu F, Wang S, Agbodo N, Freeman VL (2020). Rapidly measuring spatial accessibility of COVID-19 healthcare resources: a case study of Illinois, USA. Int J Health Geogr.

[37] Leung C (2020). Risk factors for predicting mortality in elderly patients with COVID-19: A review of clinical data in China. Mech Ageing Dev.

[38] MohammadEbrahimi S, Mohammadi A, Bergquist R, Dolatkhah F, Olia M, Tavakolian A (2021). Epidemiological characteristics and initial spatiotemporal visualisation of COVID-19 in a major city in the Middle East. BMC Public Health.

[39] Shabanikiya H, Hashtarkhani S, Bergquist R, Bagheri N, Vafaeinejad R, Amiri-Gholanlou M (2020). Multiple-scale spatial analysis of paediatric, pedestrian road traffic injuries in a major city in North-Eastern Iran 2015–2019. BMC Public Health.

[40] Centers for Disease Control and Prevention. Risk for COVID-19 infection. Hospitalization, and death by age group. https://www.cdc.gov/coronavirus/2019-ncov/downloads/covid-data/hospitalization-death-by-age.pdf. Accessed 25 Jun 2021.

[41] Masoodi M, Rahimzadeh M (2015). Measuring access to urban health services using Geographical Information System (GIS): a case study of health service management in Bandar Abbas, Iran. Int J Health Policy Manag.

[42] Dadashpoor H, Rostami F, Alizadeh B (2016). Is inequality in the distribution of urban facilities inequitable? Exploring a method for identifying spatial inequity in an Iranian city. Cities.

[43] Wiśniewski S (2016). Spatial accessibility of hospital healthcare in Łódź voivodeship. Quaest Geogr.

[44] Wan N, Zou B, Sternberg T (2012). A three-step floating catchment area method for analyzing spatial access to health services. Int J Geogr Inf Syst.

[45] Sarani S (2011). Measuring physical accessibility to health facilities—a case study on Khulna City. World Health Popul..

[46] Parr JB (1980). Health care facility planning: some developmental considerations. Socio-Econ Plan Sci.

[47] Cheng G, Zeng X, Duan L, Lu X, Sun H, Jiang T (2016). Spatial difference analysis for accessibility to high level hospitals based on travel time in Shenzhen. China Habitat Int.

[48] Silalahi FES, Hidayat F, Dewi RS, Purwono N, Oktaviani N (2020). GIS-based approaches on the accessibility of referral hospital using network analysis and the spatial distribution model of the spreading case of COVID-19 in Jakarta, Indonesia. BMC Health Serv Res.

[49] Pugh S, editor. Concept selection: a method that works. Review of design methodology. Proceedings international conference on engineering design; 1981; Rome: Heurista.

[50] Burge S. The systems engineering tool box. 2006. https://www.burgehugheswalsh.co.uk/Uploaded/1/Documents/Needs-Means-Analysis-Tool-v1.pdf. Accessed 25 Jun 2021.

[51] Chirisa I, Mutambisi T, Chivenge M, Mabaso E, Matamanda AR, Ncube R (2020). The urban penalty of COVID-19 lockdowns across the globe: manifestations and lessons for Anglophone sub-Saharan Africa. GeoJournal.

[52] Goepel KD (2018). Implementation of an online software tool for the analytic hierarchy process (AHP-OS). Int J Anal Hierarchy Process..

[53] Anselin L (1995). Local indicators of spatial association—LISA. Geogr Anal.

[54] Pishgar E, Fanni Z, Tavakkolinia J, Mohammadi A, Kiani B, Bergquist R (2020). Mortality rates due to respiratory tract diseases in Tehran, Iran during 2008–2018: a spatiotemporal, cross-sectional study. BMC Public Health.

[55] Yamada I, Richardson D, Castree N, Goodchild MF, Kobayashi A, Liu W, Marston RA (2016). Thiessen polygons. International encyclopedia of geography: people, the earth, environment and technology.

[56] Jacobson SH, Sewell EC, Proano RA (2006). An analysis of the pediatric vaccine supply shortage problem. Health Care Manag Sci.

[57] Shadmi E, Chen Y, Dourado I, Faran-Perach I, Furler J, Hangoma P (2020). Health equity and COVID-19: global perspectives. Int J Equity Health.

[58] Zhao P, Li S, Liu D (2020). Unequable spatial accessibility to hospitals in developing megacities: New evidence from Beijing. Health Place.

[59] Kenyon S, Lyons G, Rafferty J (2002). Transport and social exclusion: investigating the possibility of promoting inclusion through virtual mobility. J Transp Geogr.

[60] Tseng M-H, Wu H-C (2021). Accessibility assessment of community care resources using maximum-equity optimization of supply capacity allocation. Int J Environ Res Public Health.

