# Supplementary material for: Measuring COVID-19 vaccination coverage: an enhanced age-adjusted two-step floating catchment area model
Source: Infect Dis Poverty. 2021 Sep 16;10:118. doi: 10.1186/s40249-021-00904-6 (PMC8443959; doi:10.1186/s40249-021-00904-6)
Supplement: Supplementary file 1 — Additional file 1. The flowchart view of the methodology scheme in this study. [file 40249_2021_904_MOESM1_ESM.pdf]

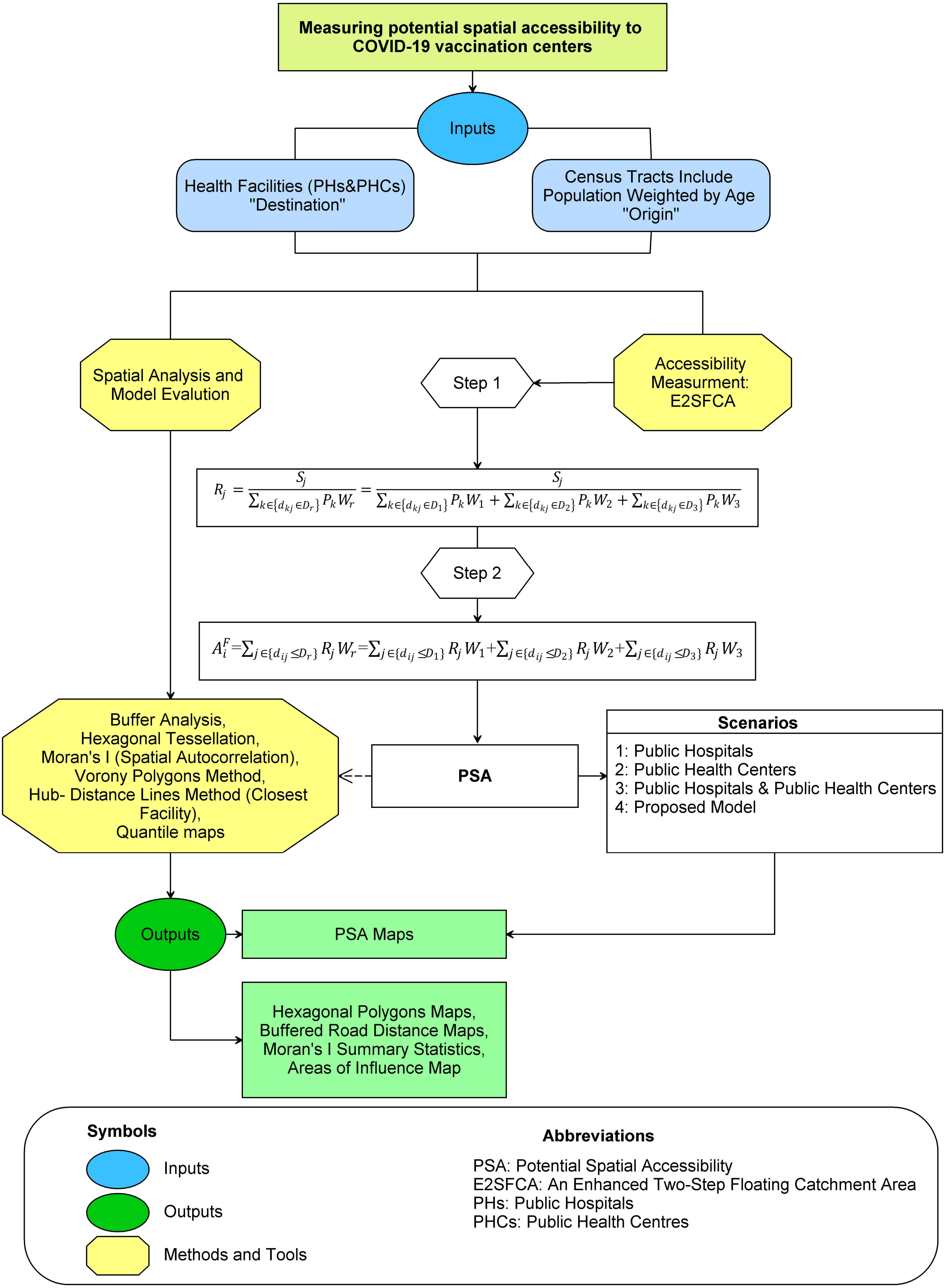

Measuring potential spatial accessibility to COVID-19 vaccination centers
Inputs
Health Facilities (PHs&PHCs) "Destination"
Census Tracts Include Population Weighted by Age "Origin"
Spatial Analysis and Model Evalution
Step 1
Accessibility Measurment: E2SFCA
$R_j = \frac{S_j}{\sum_{k\in\{d_{kj}\in D_r\}} P_k W_r} = \frac{S_j}{\sum_{k\in\{d_{kj}\in D_1\}} P_k W_1 + \sum_{k\in\{d_{kj}\in D_2\}} P_k W_2 + \sum_{k\in\{d_{kj}\in D_3\}} P_k W_3}$
Step 2
$A_i^F = \sum_{j\in\{d_{ij}\leq D_r\}} R_j W_r = \sum_{j\in\{d_{ij}\leq D_1\}} R_j W_1 + \sum_{j\in\{d_{ij}\leq D_2\}} R_j W_2 + \sum_{j\in\{d_{ij}\leq D_3\}} R_j W_3$
Buffer Analysis, Hexagonal Tessellation, Moran's I (Spatial Autocorrelation), Vorony Polygons Method, Hub- Distance Lines Method (Closest Facility), Quantile maps
PSA
Scenarios
1: Public Hospitals
2: Public Health Centers
3: Public Hospitals & Public Health Centers
4: Proposed Model
Outputs
PSA Maps
Hexagonal Polygons Maps, Buffered Road Distance Maps, Moran's I Summary Statistics, Areas of Influence Map
Symbols
Inputs
Outputs
Methods and Tools
Abbreviations
PSA: Potential Spatial Accessibility
E2SFCA: An Enhanced Two-Step Floating Catchment Area
PHs: Public Hospitals
PHCs: Public Health Centres
